# Five-Year Drug Survival and Discontinuation Reasons for Eight Biological Disease-Modifying Antirheumatic Drugs for Rheumatoid Arthritis: A Retrospective Analysis of 1182 Patients from the Niigata Orthopedic Surgery Rheumatoid Arthritis Database (NOSRAD)

**DOI:** 10.3390/jcm15052075

**Published:** 2026-03-09

**Authors:** Nariaki Hao, Naoki Kondo, Katsumitsu Arai, Naoko Kudo, Takehiro Murai, Junichi Fujisawa, Yasufumi Kijima, Rika Kakutani, Hiroyuki Kawashima

**Affiliations:** 1 Division of Orthopedic Surgery, Department of Regenerative and Transplant Medicine, Graduate School of Medical and Dental Sciences, Niigata University, Niigata 951-8510, Japan; nariaki_hao@yahoo.co.jp (N.H.); m05039sab@yahoo.co.jp (Y.K.); kakutani1115@fine.ocn.ne.jp (R.K.); inskawa@med.niigata-u.ac.jp (H.K.); 2 Department of Orthopedic Surgery, Niigata Prefectural Central Hospital, 205 Shinnan-cho, Joetsu-shi, Niigata 943-0192, Japan; araika231@zmail.plala.or.jp; 3 Katsumi Seikeigeka Clinic, 1-27-12 Toyano, Chuo-ku, Niigata 950-0951, Japan; naokokudo1973@gmail.com; 4 Murai Seikeigeka Clinic, 1-3-6 Kamikido, Higashi-ku, Niigata 950-0891, Japan; muraiseikei@ray.ocn.ne.jp; 5 Department of Orthopedic Surgery, NHO Nishiniigata Chuo Hospital, 1-14-1 Masago, Nishi-ku, Niigata 950-2085, Japan; jfujisawa@me.com

**Keywords:** arthritis, rheumatoid, antirheumatic agents, biological therapy, medication adherence, treatment failure, drug-related side effects and adverse reactions, retrospective studies, cohort studies, registries, Japan

## Abstract

**Background**: Continuity of care for rheumatoid arthritis patients within regional networks enables stable long-term clinical data collection, despite chronic rheumatologist shortages in Japan. We determined 5-year drug survival and discontinuation reasons for eight biological disease-modifying antirheumatic drugs (bDMARDs) using a regional multicenter registry. **Methods**: We retrospectively analyzed 1182 patients initiating their first (naïve, *n* = 784) or subsequent (switch, *n* = 398) bDMARD between May 2001 and August 2022 across five institutions. The primary endpoint (5-year drug survival) and secondary endpoints (discontinuation risk factors and cumulative incidence of reasons) were evaluated using Kaplan–Meier curves, Cox proportional hazards, and Fine & Gray models. **Results**: Baseline characteristics varied significantly among bDMARDs. Five-year drug survival in the naïve cohort ranged from tocilizumab (50.8%) to golimumab (22.6%); in the switch cohort, from abatacept (42.6%) to infliximab (10.0%). In multivariable Cox analysis of naïve patients, male sex (hazard ratio [HR] = 1.49, 95% confidence interval [CI] = 1.09–2.02), lower baseline 28-joint Disease Activity Score with erythrocyte sedimentation rate (DAS28-ESR) (HR = 0.90, 95% CI = 0.82–0.99), and absence of methotrexate co-therapy (HR = 0.73, 95% CI = 0.55–0.97) predicted discontinuation. The lower baseline DAS28-ESR association potentially reflects successful courses toward intentional cessation following remission. Discontinuations were attributed to inadequate response (27.1%), non-adverse events (25.3%), and adverse events (17.3%). **Conclusions**: Tocilizumab and abatacept demonstrated the highest retention rates in biologic-naïve and switch cohorts, respectively. Early, individualized drug selection and dose optimization are crucial to maximizing long-term bDMARD effectiveness before switching.

## 1. Introduction

Rheumatoid arthritis (RA), a systemic autoimmune disease characterized by synovial inflammation, culminates in progressive joint destruction [1]. The prevalence of RA varies from approximately 0.2–0.5% among many nations [2]. Recent advances in the elucidation of the immunological mechanisms involved in the onset and progression of RA—particularly the roles of pro-inflammatory cytokines—have substantially deepened our understanding of its pathophysiology [3,4].

In Japan, biological disease-modifying antirheumatic drugs (bDMARDs) were introduced in 2003, ushering in a paradigm shift in RA management [5,6,7]. Although bDMARDs exhibit high efficacy, their drug survival and the reasons for discontinuation may be influenced by multiple factors, including patient characteristics, regional contexts, and health-care delivery systems. While numerous multicenter studies have addressed this topic [8,9], there remains a need for long-term, consistent real-world data from regional multicenter networks, reflecting specialized treatment strategies in specific clinical settings [10].

Continuity of care within a coordinated regional network of specialized institutions reduces outpatient attrition and enables a stable collection of long-term clinical data. However, our prefecture, like many regional areas in Japan, faces a chronic shortage of board-certified rheumatologists [11,12]. This scarcity of specialized healthcare professionals poses a significant challenge to consistent patient management and equitable access to advanced therapies. To overcome these limitations, optimizing the management of healthcare resources and fostering community-level medical collaboration are essential. Indeed, the recent literature emphasizes that effective management and strategic community involvement within orthopedic and specialized healthcare domains are crucial for sustaining high-quality patient care amidst workforce constraints [13]. Addressing these systemic challenges requires robust real-world data from regional networks. Therefore, in this study, we utilized the Niigata Orthopedic Surgery Rheumatoid Arthritis Database (NOSRAD), a multicenter registry, to retrospectively analyze the drug survival and discontinuation reasons of eight bDMARDs administered to patients with RA within this regional network.

## 2. Materials and Methods

### 2.1. Study Design, Setting, and Population

The NOSRAD is an ongoing, observational, multicenter registry that prospectively collects clinical data on every patient with RA treated at five participating institutions in Niigata, Japan: the Department of Orthopedic Surgery at Niigata University, Niigata Prefectural Central Hospital, Katsumi Seikeigeka Clinic, Murai Seikeigeka Clinic, and NHO Nishiniigata Chuo Hospital. The registry systematically captures longitudinal data, including patient demographics, disease activity indices (e.g., DAS28-ESR), detailed medication history (start/stop dates and reasons for discontinuation), laboratory findings, and adverse events. Data entry is performed by the treating rheumatologists during routine clinical visits, ensuring high clinical accuracy and completeness. For this retrospective cohort study, we screened all consecutive patients registered in NOSRAD between 1 May 2001 and 31 August 2022 (*n* = 1517). Regarding the handling of missing data, we employed a complete-case analysis approach; to maintain the robustness of our multivariable models, patients with missing values for any key baseline variable (such as DAS28-ESR or concomitant medication status) were strictly excluded from the analytic cohort. The date of initiating each bDMARD was defined as the index date, and all clinical and demographic data assessed at that time point were considered as baseline data.

#### 2.1.1. Inclusion Criteria

(i) Age ≥18 years at the index date.
(ii) Fulfilment of either the 1987 revised American College of Rheumatology (ACR) criteria or the 2010 ACR/European League Against Rheumatism (EULAR) classification criteria for RA [14,15].
(iii) Receipt of at least one approved dose of any of the following eight originator bDMARDs: infliximab (IFX; Mitsubishi Tanabe Pharma, Osaka, Japan), etanercept (ETN; Pfizer Japan, Tokyo, Japan), tocilizumab (TCZ; Chugai Pharmaceutical, Tokyo, Japan), adalimumab (ADA; AbbVie, North Chicago, IL, USA), abatacept (ABT; Ono Pharmaceutical, Osaka, Japan), golimumab (GLM; Janssen Pharmaceutical, Beerse, Belgium), certolizumab pegol (CZP; Astellas Pharma, Tokyo, Japan) or sarilumab (SAR; Asahi Kasei Pharma, Tokyo, Japan). Biosimilar agents were not included in this study. Intravenous and subcutaneous formulations were analyzed together as a single group for each drug to maintain sufficient statistical power.
(iv) Complete data on baseline demographics, disease characteristics, previous conventional synthetic DMARD (csDMARD) exposure, and comorbidities.
(v) At least one follow-up visit recorded after the index bDMARD administration.

#### 2.1.2. Exclusion Criteria

(i) Current treatment using any targeted synthetic DMARD (*n* = 230);
(ii) Diagnosis of spondyloarthritis or other inflammatory arthritides (*n* = 14);
(iii) Missing values on any key variable (*n* = 91);
(iv) Off-label bDMARD dosing or single, unconfirmed exposure lasting <1 month.

Patients who initiated their first bDMARD were defined as the naïve cohort, and those who switched from at least one prior bDMARD were defined as the switch cohort. Follow-up continued from the first documented dose until permanent drug discontinuation, death, loss to follow-up (>12 months without contact), or 31 August 2022, whichever occurred first.

### 2.2. Treatment and Follow-Up

Therapy was administered in accordance with the clinical practice guidelines of the Japan College of Rheumatology and was managed by six board-certified rheumatologists (NK, KA, NK, TM, JF, and KY). After bDMARD initiation, patients attended the orthopedic outpatient clinic every 4–12 weeks. Dose changes in bDMARDs, MTX, or PSL during the follow-up period were not recorded in the registry; therefore, baseline doses were strictly used for all analyses.

### 2.3. Outcome Measures

The primary endpoint was the 5-year drug survival rate for each bDMARD. Drug survival was defined retrospectively as the interval from the first administration of a bDMARD to permanent discontinuation. Discontinuation was directly ascertained and recorded in the NOSRAD registry by the treating board-certified rheumatologists at the time the clinical decision was made, rather than being inferred from prescription refill gaps (i.e., no grace period was used). The date of discontinuation was defined as the date when the physician explicitly documented the cessation of the drug along with its primary reason. Secondary endpoints included the identification of baseline factors associated with treatment discontinuation over the 5-year follow-up period. Additionally, we performed stratified analyses to compare 5-year drug survival according to concomitant methotrexate (MTX) use at baseline.

### 2.4. Explanatory Variables

Candidate variables were sex, age, disease duration, baseline 28-joint Disease Activity Score using erythrocyte sedimentation rate (DAS28-ESR), concomitant MTX, and concomitant prednisolone (PSL). Reasons for discontinuation were categorized as inadequate response (including primary and secondary); adverse events (infection, pulmonary, liver, skin disorders, cardiovascular disease, malignant tumor, and others); or non-adverse events (remission or good response, patient preference, transfer to another hospital, death, other reasons, and unknown). Physicians were allowed to cite only one reason for the discontinuation.

### 2.5. Statistical Analysis

Baseline characteristics were summarized using descriptive statistics. Categorical variables were compared using the chi-square test; continuous variables were compared using the Kruskal–Wallis test, with multiple comparisons adjusted by the Steel–Dwass method. Drug survival curves were estimated by the Kaplan–Meier method and compared with the log-rank test. Cumulative incidence functions for each discontinuation reason were derived from a competing-risks model and compared with the Fine & Gray test.

We used Cox proportional hazards model, stratified by drug type, to analyze factors influencing treatment discontinuation. Explanatory variables were sex, age, disease duration, DAS28-ESR, and concomitant use of MTX and PSL. The proportional hazards assumption was assessed with Schoenfeld residuals. Bonferroni adjustment was applied to control the family-wise error rate arising from multiple comparisons.

All statistical analyses were performed by using EZR software version 1.68 (Saitama Medical Centre, Jichi Medical University, Saitama, Japan) [16], which is a graphical user interface for R version 4.4.1 (The R Foundation for Statistical Computing, Vienna, Austria). A two-sided *p* value < 0.05 was considered statistically significant.

## 3. Results

### 3.1. Baseline Characteristics

**Figure 1:**
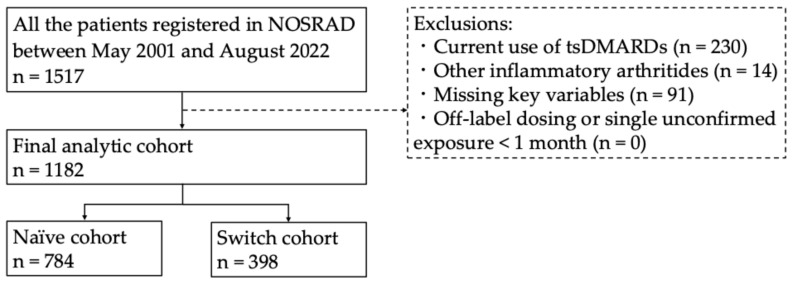
Flowchart of the patient selection process.

A flowchart of the patient selection process is shown in Figure 1. After the exclusions, the final analytic cohort comprised 1182 patients. Patients who initiated their first bDMARD constituted the naïve cohort (*n* = 784), and those who switched from at least one prior bDMARD formed the switch cohort (*n* = 398). Baseline characteristics of the naïve cohort (*n* = 784) are summarized in Table 1. Significant inter-drug differences were observed for sex, age, disease duration, DAS28-ESR, concomitant MTX use, concomitant PSL use, and PSL dose (*p* = 0.040, *p* < 0.001, *p* < 0.001, *p* < 0.001, *p* < 0.001, *p* = 0.002, and *p* = 0.012, respectively; Kruskal–Wallis test). The mean age of patients receiving ABT was 70.4 years, significantly higher than that of patients treated with IFX (55.1 years; *p* < 0.001), ETN (56.4 years; *p* = 0.002), or TCZ (56.7 years; *p* = 0.006), according to the Steel–Dwass test. MTX dose did not differ among the patients regardless of the drugs.

Baseline characteristics of the switch cohort (*n* = 398) are shown in Table 2. Significant differences were found among the patients based on the drugs in age, disease duration, DAS28-ESR, concomitant MTX use, PSL dose, and use as a second- or fourth-line agent (*p* < 0.001, *p* = 0.020, *p* = 0.002, *p* < 0.001, *p* = 0.032, *p* = 0.006, and *p* = 0.028, respectively; Kruskal–Wallis test). No significant differences were detected in sex, MTX dose, concomitant PSL use, or use as a third-line agent. The mean age of ABT-treated patients was 70.1 years, significantly exceeding that of patients receiving ETN (55.1 years; *p* < 0.001); TCZ (59.2 years; *p* < 0.001); ADA (54.6 years; *p* < 0.001); or CZP (56.3 years; *p* = 0.010), according to the Steel–Dwass test. GLM users were also older (67.3 years) than those on ETN (55.1 years; *p* = 0.019) or ADA (54.6 years; *p* = 0.047), according to the Steel–Dwass test.

In the overall cohort (*n* = 1182), significant inter-drug differences were detected in age, disease duration, DAS28-ESR, concomitant MTX use, concomitant PSL use, PSL dose, and use as first-, second-, third-, or fourth-line therapy (all *p* < 0.001; however, PSL use *p* = 0.005 and PSL dose *p* = 0.001; Kruskal–Wallis test), whereas sex and MTX dose did not differ (Table S1). The mean age of ABT users was 70.3 years, significantly higher than that of patients treated with IFX (55.6 years; *p* = 0.004); ETN (56.1 years; *p* < 0.001); TCZ (57.6 years; *p* < 0.001); or ADA (56.9 years; *p* = 0.040), according to the Steel–Dwass test.

### 3.2. Drug Survival Rates

**Figure 2:**
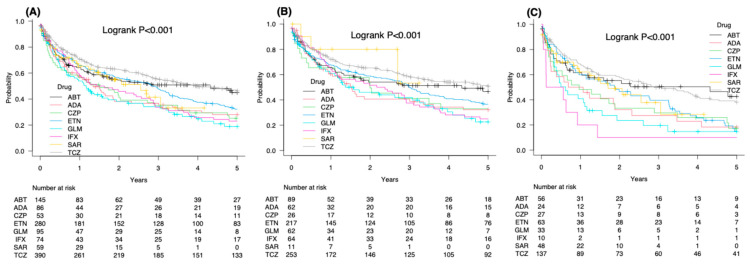
Kaplan–Meier drug survival curves for each bDMARD: (**A**) all 1182 patients, (**B**) 784 naïve patients, and (**C**) 398 switch patients.

In the overall cohort, TCZ showed the highest 5-year drug survival (46.3%), followed by ABT (45.0%), ETN (32.2%), ADA (28.0%), CZP (25.2%), IFX (23.0%), and GLM (18.9%) (Figure 2A). TCZ survival was significantly superior to that of IFX, ETN, ADA, GLM, and CZP (all *p* ≤ 0.001, Bonferroni-adjusted log-rank test). No significant difference was observed between TCZ and ABT.

In the naïve cohort, TCZ again demonstrated the highest 5-year survival (50.8%), followed by ABT (46.6%), ETN (36.6%), CZP (33.0%), ADA (32.3%), IFX (25.0%), and GLM (22.6%) (Figure 2B). TCZ survival exceeded that of IFX, ETN, and GLM (all *p* < 0.001, Bonferroni-adjusted log-rank test).

In the switch cohort, ABT achieved the best 5-year survival (42.6%), followed by TCZ (38.2%), ADA (18.3%), ETN (15.1%), CZP (17.3%), GLM (14.8%), and IFX (10.0%) (Figure 2C). TCZ survival was higher than that of IFX and GLM (both *p* < 0.001, Bonferroni-adjusted log-rank test), and ABT survival was higher than IFX (*p* < 0.001, Bonferroni-adjusted log-rank test).

**Figure 3:**
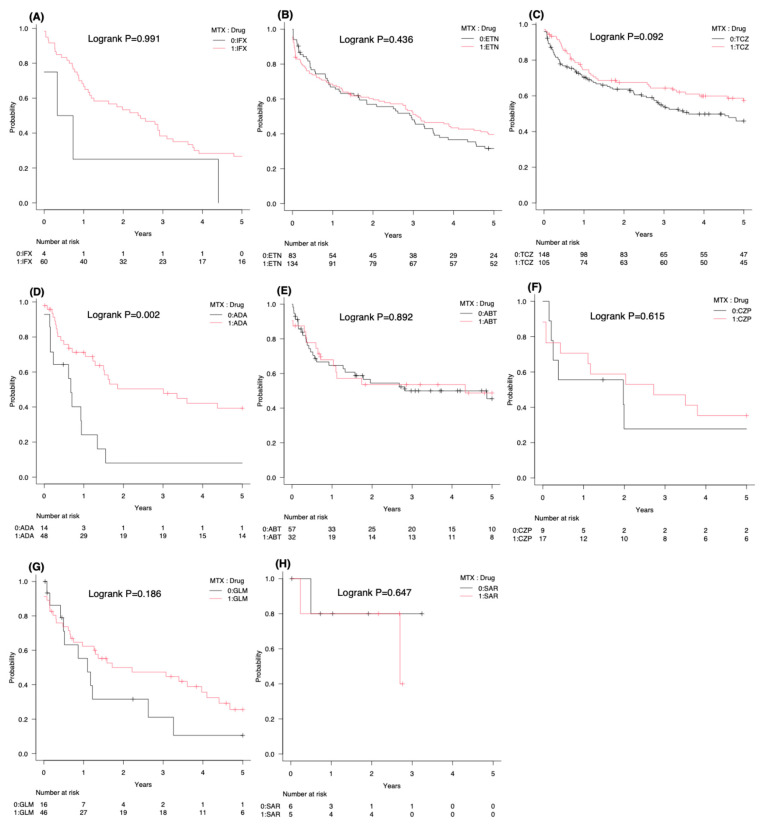
Kaplan–Meier drug survival curves stratified by MTX co-treatment status (0 = without MTX, 1 = with MTX) in naïve patients for each bDMARD: (**A**) IFX, (**B**) ETN, (**C**) TCZ, (**D**) ADA, (**E**) ABT, (**F**) CZP, (**G**) GLM, and (**H**) SAR. MTX status (0 or 1) is indicated in the legends of each graph. Log-rank *p*-values are provided in each panel.

Stratified analyses revealed generally better survival in MTX-combination therapy than in monotherapy (Figures S1 and S2). Notably, in the naïve cohort, ADA survival was significantly higher with MTX combination therapy (*p* < 0.002, Bonferroni-adjusted log-rank test) (Figure 3).

### 3.3. Risk Factors for Drug Discontinuation

In the Cox proportional hazards model stratified by drug type (strata), significant predictors of discontinuation in the naïve cohort were: sex (male vs. female: hazard ratio [HR] = 1.49, 95% confidence interval [CI] = 1.09–2.02, *p* = 0.011); baseline DAS28-ESR (per unit decrease: HR = 0.90, 95% CI = 0.82–0.99, *p* = 0.039); and concomitant MTX use (yes vs. no: HR = 0.73, 95% CI = 0.55–0.97, *p* = 0.028) (Table 3).

### 3.4. Reasons for Drug Discontinuation

Overall, 823 patients (69.6%) discontinued bDMARD. The reasons were inadequate response (*n* = 320, 27.1%); adverse events (*n* = 204, 17.3%); and non-adverse events (*n* = 299, 25.3%) (Table 4). Adverse events comprised infections (*n* = 79, 6.7%); pulmonary disorders (*n* = 12, 1.0%); liver disorders (*n* = 9, 0.8%); skin disorders (*n* = 28, 2.4%); cardiovascular disease (*n* = 7, 0.6%); malignant tumor (*n* = 15, 1.3%); and others (*n* = 54, 4.6%). Non-adverse events included remission or good response (*n* = 43, 3.6%); patient preference (*n* = 33, 2.8%); transfer to another hospital (*n* = 167, 14.1%); other reasons (*n* = 11, 0.9%); and unknown (*n* = 45, 3.8%) (Table S4)

The drug-by-drug comparison revealed a significant difference in discontinuations attributable to inadequate response (*p* < 0.001, chi-square test). The proportions were highest for CZP (*n* = 22, 41.5%); IFX (*n* = 30, 40.5%); and ADA (*n* = 33, 38.4%); followed by ETN (*n* = 96, 34.3%); GLM (*n* = 27, 28.4%); SAR (*n* = 14, 23.7%); ABT (*n* = 33, 22.8%); and TCZ (*n* = 65, 16.7%).

The absolute numbers and percentages of discontinuations of the following drugs due to adverse events were: CZP (*n* = 13, 24.5%), IFX (*n* = 15, 20.3%), ETN (*n* = 56, 20.0%), ADA (*n* = 15, 17.4%), GLM (*n* = 16, 16.8%), TCZ (*n* = 65, 16.7%), ABT (*n* = 19, 13.1%), and SAR (*n* = 5, 8.5%). Although a global chi-square test did not reach significance level, the distribution indicates relatively fewer adverse-event discontinuations with SAR and ABT.

Discontinuations for non-adverse events—including remission, patient preference, and transfer to another hospital—also differed significantly among drugs (*p* < 0.001, chi-square). This was highest for ETN (*n* = 96, 34.3%), followed by IFX (*n* = 22, 29.7%), TCZ (*n* = 99, 25.4%), GLM (*n* = 24, 25.3%), ABT (*n* = 27, 18.6%), ADA (*n* = 16, 18.6%), SAR (*n* = 8, 13.6%), and CZP (*n* = 7, 13.2%).

**Figure 4:**
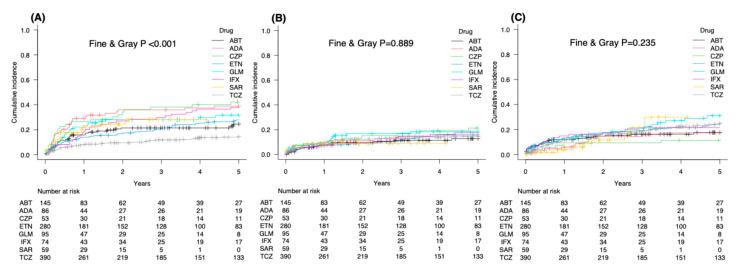
Cumulative incidence of treatment discontinuation by reason for each bDMARD: (**A**) inadequate response, (**B**) adverse events, and (**C**) non-adverse events. Cumulative incidence was estimated using the Fine & Gray competing risks model.

Competing-risk analysis demonstrated that discontinuation owing to inadequate response rose steeply during the first 2 years and more gradually thereafter (Figure 4A). At 5 years, the cumulative incidence was highest for CZP (42.2%), followed by ADA (35.9%), IFX (37.8%), GLM (28.0%), ETN (27.9%), ABT (24.5%), and lowest for TCZ (9.5%). The rate of TCZ was significantly lower than those of IFX (*p* = 0.004), ETN (*p* = 0.003), ADA (*p* < 0.001), GLM (*p* = 0.009), CZP (*p* < 0.001), and SAR (*p* = 0.026), based on Bonferroni-adjusted log-rank test (*p* < 0.001 overall, Fine & Gray test).

Discontinuations attributed to adverse events increased during the first year and then plateaued (Figure 4B). At 5 years, the cumulative incidence was highest for CZP (21.3%), followed by GLM (18.3%), IFX (17.6%), ETN (15.8%), ADA (15.7%), TCZ (14.5%), ABT (12.8%), and lowest for SAR (8.8%).

For non-adverse events, the 5-year cumulative incidence was highest for GLM (31.1%), followed by TCZ (24.8%), ETN (24.2%), IFX (21.6%), ABT (17.7%), ADA (17.4%), CZP (11.4%) (Figure 4C).

## 4. Discussion

In this large, multicenter retrospective cohort study, we systematically analyzed the 5-year drug survival and discontinuation reasons for eight bDMARDs in 1182 patients with RA in Japan. Although numerous large-scale multicenter studies and database analyses of drug persistence have been published [8,9], investigations enrolling more than 1000 patients within a specialized regional network—consisting of a university hospital, a regional central hospital, and specialized clinics—where all patients were managed by specialists in the same field (orthopedic rheumatology) with a unified treatment approach, are extremely rare [10]. This consistency within a multicenter framework is considered the key novelty of the present work.

In the overall and naïve cohorts, TCZ demonstrated the highest drug survival, and ABT also maintained favorable persistence. These trends are partly consistent with previous reports [10,17,18,19,20,21,22,23]. A notable clinical advantage of TCZ and ABT is that they remain effective without concomitant MTX, benefitting older adult patients or those unsuitable for combination therapy [20,24]. Among tumor necrosis factor inhibitors (TNFi), ETN outlasts IFX and ADA [25,26], with IFX often showing the poorest survival [25,27], similar to the pattern observed in the present study.

Notably, while the highest discontinuation rate shown by CZP is considered due to inadequate response in our cohort, baseline characteristics revealed that the CZP group comprised a high proportion of switch patients compared to other TNFi. Given that treatment efficacy is generally lower in switch cohorts than in naïve cohorts, this result likely reflects the higher proportion of prior biologic exposure in the CZP group rather than a purely drug-specific characteristic.

Conversely, certain TNFi such as IFX and GLM were often discontinued early owing to insufficient efficacy, suggesting that earlier switching to agents with different mechanisms may be beneficial. The unexpectedly high survival of ABT in the switch cohort indicates retained efficacy in refractory cases previously exposed to multiple bDMARDs, supporting mechanism-based switching strategies.

In naïve patients on ADA, concomitant MTX significantly improved survival. Monoclonal-antibody TNFi are susceptible to anti-drug antibody formation, which undermines therapeutic efficacy [28,29,30,31]. ADA is a fully human monoclonal antibody; however, it does not completely escape immunogenicity. These findings underscore the importance of MTX-mediated suppression of anti-drug antibodies [17,19,22,26,32,33,34,35,36,37,38,39,40,41].

In the naïve cohort, Cox analysis identified male sex, lower baseline DAS28-ESR, and absence of MTX co-therapy as independent predictors of discontinuation. Unlike previous studies showing poorer persistence in females [42,43,44], male sex remained a risk factor even after censoring discontinuations due to hospital transfer or patient preference (HR = 1.54, 95% CI = 1.09–2.17, *p* = 0.013). Possible explanations for the early discontinuation in males include inefficacy or adverse events, relatively lower per-kilogram dosing with fixed-dose subcutaneous regimens in individuals with higher body mass index [45,46], and enhanced immunogenicity associated with higher smoking rates [47,48]. Notably, although low baseline disease activity was identified as a factor associated with biologic discontinuation, this may represent a favorable clinical aspect—that is, discontinuation can be intentionally achieved once remission is attained. Unlike discontinuations due to therapeutic failure, higher discontinuation rates among patients with low disease activity likely reflect a successful clinical course leading to intentional cessation after achieving stable remission. However, we must explicitly acknowledge that this interpretation remains speculative. Because DAS28-ESR was only measured at baseline (treatment initiation) in our multivariable models, it does not capture the longitudinal changes in disease activity immediately preceding the physician’s decision to discontinue the drug. Further studies incorporating time-varying covariates are needed to confirm this hypothesis. As in previous studies, no MTX is a negative predictor of persistence [17,25,49,50], and low MTX dosage has likewise been implicated [8,16,24,49]. Japanese cohorts, with a mean MTX dose (7.5 ± 2.3 mg/week in this study), often benefit from lower MTX doses than Western populations [51]. PSL co-therapy had no influence on survival [52].

Although inadequate effectiveness was the leading cause of discontinuation (27.1%), adverse events (17.3%) and non-adverse events (25.3%) were also substantial. It is important to note that the “non-adverse events” category comprises highly heterogeneous reasons, ranging from positive clinical outcomes (e.g., remission or good response) to neutral administrative reasons (e.g., hospital transfer) and patient preferences (detailed in Table S4). Discontinuations due to hospital transfer (14.1%) constituted the majority of this category, which may reflect regional specialist maldistribution and referral patterns in the provincial areas. To address this heterogeneity and evaluate pure pharmacological persistence, we performed a sensitivity analysis wherein hospital transfers were censored. This analysis confirmed that our primary findings regarding the inter-drug survival trends remained robust (Figure S3, Tables S5–S7). ABT had the lowest adverse-event discontinuation rate in our cohort, consistent with some previous reports of fewer episodes of severe infections and infusion reactions [17,53,54]. However, this finding should be interpreted cautiously. Larger real-world studies in older populations have demonstrated heterogeneous persistence patterns across biologics, identifying advanced age and polypharmacy—rather than specific molecules alone—as key predictors of treatment discontinuation [55]. Therefore, while ABT remains a reasonable option for older adults, individualized risk assessment including comorbidities and polypharmacy is crucial.

Inadequate response discontinuations clustered within 2 years of initiation, while adverse events peaked in the first year, highlight the first two years as critical for bDMARD success and underscore the necessity for a treat-to-target strategy with timely assessment and switching [56].

This study has several limitations. First, although this is a multicenter study, it is a retrospective analysis of observational registry data from a specific geographic region in Japan. Consequently, selection bias, information bias, and limited generalizability to other regions or different medical specialties (e.g., internal medicine rheumatology) cannot be excluded. Second, discontinuation decisions and explanations were clinician-dependent, not standardized. Third, baseline characteristics differed among drugs, and unmeasured confounding may persist despite adjustment. Fourth, minor dose modifications of bDMARDs, MTX, or PSL were not captured. Fifth, differences between intravenous and subcutaneous preparations and concomitant csDMARDs could not be fully ascertained. Sixth, CZP and SAR were approved relatively recently in Japan (2013 and 2017, respectively), explaining the smaller sample sizes that may have affected the estimates. Seventh, we evaluated RA disease activity primarily using DAS28-ESR. Although CDAI and SDAI have shown higher precision for defining clinical remission [57], they were not included in this analysis because the necessary components for their calculation were not consistently available in our database, particularly in the earlier years of the registry in 2001. To maintain data consistency across the two-decade study period, DAS28-ESR was utilized as the primary measure of disease activity. Eighth, this study exclusively analyzed originator bDMARDs and excluded biosimilar agents. Given that biosimilars are currently integrated into standard clinical practice due to their cost-effectiveness, our findings regarding drug survival and discontinuation reasons may not fully generalize to contemporary treatment settings where biosimilars are frequently prescribed. Ninth, our study spanned over two decades (2001–2022). During this extensive period, treatment strategies for RA evolved substantially, most notably with the widespread adoption of the treat-to-target (T2T) strategy, and the availability of various bDMARDs changed. Because recently approved agents inherently have shorter observation periods, adjusting our survival analyses for the calendar year of treatment initiation would cause severe statistical fragmentation. Consequently, we could not adjust for calendar year, and this evolving historical context may have acted as an unmeasured confounder in our estimates.

Despite these constraints, this study provides high-resolution real-world evidence through long-term follow-up and detailed analysis of discontinuation reasons within a coordinated regional multicenter network. The consistent management by specialists across diverse clinical settings—from a university hospital to specialized clinics—strengthens the clinical relevance of our findings regarding drug survival and predictors of treatment cessation. Future large-scale, prospective studies—incorporating more stringent disease activity measures such as CDAI and SDAI, as well as immunological and biomarker data—are essential to further validate these results and refine personalized therapeutic strategies for the long-term management of RA.

## 5. Conclusions

In this multicenter, long-term retrospective cohort of 1182 patients with RA, we compared 5-year drug survival and discontinuation reasons for eight bDMARDs. TCZ achieved the highest survival in biologic-naïve patients, driven by a remarkably low rate of discontinuation due to inadequate response, whereas ABT ranked first among the switch patients and demonstrated robust retention even in refractory cases. Transitioning from one bDMARD to another markedly reduced persistence overall. ABT—used more frequently in older patients—exhibited a comparatively low rate of adverse-event discontinuations, underscoring its clinical utility in older adult populations. Concomitant MTX significantly improved the survival of ADA. Male sex and the absence of MTX co-therapy emerged as potential risk factors for treatment cessation. While low baseline disease activity was also associated with a higher rate of discontinuation, this likely reflects a favorable clinical course leading to intentional treatment cessation after achieving remission, rather than therapeutic failure. Inadequate response was the leading cause of discontinuation, clustering within the first 2 years after initiation.

## Figures and Tables

**Table 1:** Baseline characteristics of naïve patients (*n* = 784).

	IFX (*n* = 64)	ETN (*n* = 217)	TCZ (*n* = 253)	ADA (*n* = 62)	ABT (*n* = 89)	GLM (*n* = 62)	CZP (*n* = 26)	SAR (*n* = 11)	*p*-Value
Women, *n* (%)	51 (79.7)	183 (84.3)	194 (76.7)	43 (69.4)	69 (77.5)	54 (87.1)	20 (76.9)	6 (54.5)	0.040
Age (years), mean ± SD	55.1 ± 14.9	56.4 ± 15.1	56.7 ± 16.3	57.8 ± 13.3	70.4 ± 10.5	67.4 ± 13.2	56.8 ± 14.0	68.6 ± 9.3	<0.001
Disease duration (years), mean ± SD	9.8 ± 10.0	10.5 ± 9.6	7.1 ± 9.1	10.9 ± 10.5	11.2 ± 12.5	14.6 ± 12.0	7.6 ± 7.5	16.0 ± 16.0	<0.001
DAS28-ESR, mean ± SD	4.22 ± 1.29	3.89 ± 1.31	3.74 ± 1.50	3.28 ± 1.19	4.65 ± 1.39	2.93 ± 0.76	2.39 ± 0.86	3.62 ± 0.94	<0.001
MTX use, *n* (%)	59 (92.2)	134 (62.2)	104 (41.1)	48 (77.4)	32 (36.0)	47 (67.7)	17 (65.4)	6 (54.5)	<0.001
MTX dose (mg/week), mean ± SD	7.4 ± 1.8	7.2 ± 2.1	7.7 ± 2.2	8.1 ± 2.5	7.3 ± 3.2	7.9 ± 2.3	7.4 ± 2.3	6.7 ± 0.9	0.248
PSL use, *n* (%)	28 (43.8)	113 (52.1)	97 (38.3)	22 (35.5)	54 (60.7)	29 (46.8)	10 (38.5)	7 (63.6)	0.002
PSL dose (mg/day), mean ± SD	7.9 ± 9.5	6.9 ± 5.5	8.0 ± 8.2	5.2 ± 2.6	5.7 ± 3.4	4.2 ± 2.1	6.5 ± 2.6	6.1 ± 2.9	0.012

IFX, infliximab; ETN, etanercept; TCZ, tocilizumab; ADA, adalimumab; ABT, abatacept; GLM, golimumab; CZP, certolizumab pegol; SAR, sarilumab; DAS28-ESR, the 28-joint Disease Activity Score with erythrocyte sedimentation rate; MTX, methotrexate; PSL, prednisolone; SD, standard deviation.

**Table 2:** Baseline characteristics of the switch patients (*n* = 398).

	IFX (*n* = 10)	ETN (*n* = 63)	TCZ (*n* = 137)	ADA (*n* = 24)	ABT (*n* = 56)	GLM (*n* = 33)	CZP (*n* = 27)	SAR (*n* = 48)	*p*-Value
Women, *n* (%)	6 (60.0)	53 (84.1)	111 (81.0)	17 (70.8)	48 (85.7)	23 (69.7)	24 (88.9)	37 (77.1)	0.219
Age (years), mean ± SD	58.7 ± 15.7	55.1 ± 16.3	59.2 ± 14.8	54.6 ± 14.5	70.1 ± 12.9	67.3 ± 14.0	56.3 ± 14.8	61.6 ± 14.9	<0.001
Disease duration (years), mean ± SD	7.0 ± 5.7	12.5 ± 9.5	12.4 ± 9.9	10.8 ± 8.1	16.0 ± 11.3	16.5 ± 9.3	10.1 ± 10.4	11.4 ± 7.8	0.020
DAS28-ESR, mean ± SD	4.50 ± 1.38	3.57 ± 1.38	3.37 ± 1.16	3.35 ± 1.16	4.50 ± 1.32	3.03 ± 1.32	2.94 ± 1.25	3.64 ± 1.54	0.002
MTX use, *n* (%)	9 (90.0)	43 (68.3)	77 (56.2)	19 (79.2)	22 (39.3)	22 (66.7)	13 (48.1)	19 (39.6)	<0.001
MTX dose (mg/week), mean ± SD	8.2 ± 2.0	7.6 ± 1.7	7.5 ± 2.5	7.9 ± 2.2	6.8 ± 2.5	6.5 ± 3.1	7.8 ± 2.1	7.0 ± 3.1	0.296
PSL use, *n* (%)	6 (60.0)	29 (46.0)	63 (46.0)	13 (54.2)	30 (53.6)	21 (63.6)	11 (40.7)	25 (52.1)	0.517
PSL dose (mg/day), mean ± SD	5.7 ± 3.3	5.5 ± 3.4	7.2 ± 4.9	5.0 ± 2.1	7.3 ± 7.1	5.0 ± 2.8	3.8 ± 1.2	5.2 ± 3.5	0.032
2nd bio, *n* (%)	2 (20.0)	34 (54.0)	90 (65.7)	10 (41.7)	32 (57.1)	23 (69.7)	13 (48.1)	20 (41.7)	0.006
3rd bio, *n* (%)	3 (30.0)	16 (25.4)	29 (21.2)	7 (29.2)	13 (23.2)	5 (15.2)	5 (18.5)	15 (31.3)	0.724
≧4th bio, *n* (%)	5 (50.0)	13 (20.6)	18 (13.1)	7 (29.2)	11 (19.6)	5 (15.2)	9 (33.3)	13 (27.1)	0.028

IFX, infliximab; ETN, etanercept; TCZ, tocilizumab; ADA, adalimumab; ABT, abatacept; GLM, golimumab; CZP, certolizumab pegol; SAR, sarilumab; DAS28-ESR, the 28-joint Disease Activity Score with erythrocyte sedimentation rate; MTX, methotrexate; PSL, prednisolone; bio, biologic agent; SD, standard deviation.

**Table 3:** Multivariable Cox proportional hazards model for treatment discontinuation in naïve patients, stratified by drug.

Multivariable	HR	95% CI	*p*-Value
Sex	1.49	1.09–2.02	0.011
Age	1.00	0.99–1.01	0.638
Disease duration	1.00	0.99–1.02	0.553
DAS28-ESR	0.90	0.82–0.99	0.039
MTX	0.73	0.55–0.97	0.028
PSL	1.10	0.85–1.41	0.483

*p*-values were derived from the multivariable Cox proportional hazards model, stratified by drug type. HR, hazard ratio; 95%CI, 95% confidence interval; DAS28-ESR, the 28-joint Disease Activity Score with erythrocyte sedimentation rate; MTX, methotrexate; PSL, prednisolone.

**Table 4:** Reasons for discontinuation of each bDMARD among 1182 patients.

	IFX (*n* = 74)	ETN (*n* = 280)	TCZ (*n* = 390)	ADA (*n* = 86)	ABT (*n* = 145)	GLM (*n* = 95)	CZP (*n* = 53)	SAR (*n* = 59)	*p*-Value
Inadequate response	30 (40.5)	96 (34.3)	65 (16.7)	33 (38.4)	33 (22.8)	27 (28.4)	22 (41.5)	14 (23.7)	<0.001
Adverse events	15 (20.3)	56 (20.0)	65 (16.7)	15 (17.4)	19 (13.1)	16 (16.8)	13 (24.5)	5 (8.5)	0.256
Non-adverse events	22 (29.7)	96 (34.3)	99 (25.4)	16 (18.6)	27 (18.6)	24 (25.3)	7 (13.2)	8 (13.6)	<0.001

Values are presented as *n* (percent). bDMARDs, biological disease-modifying antirheumatic drugs; IFX, infliximab; ETN, etanercept; TCZ, tocilizumab; ADA, adalimumab; ABT, abatacept; GLM, golimumab; CZP, certolizumab pegol; SAR, sarilumab.

## Data Availability

The datasets generated and/or analyzed during the current study are not publicly available due to privacy and ethical restrictions related to the nature of the clinical data. However, the data are available from the corresponding author on reasonable request, subject to the approval of the institutional ethics committee.

## Supplementary Materials

The following supporting information can be downloaded at https://www.mdpi.com/article/10.3390/jcm15052075/s1: Figure S1. Kaplan–Meier drug survival curves stratified by MTX co-treatment status (0 = without MTX, 1 = with MTX) in all patients for each bDMARD: (a) IFX, (b) ETN, (c) TCZ, (d) ADA, (e) ABT, (f) CZP, (g) GLM, and (h) SAR. MTX status (0 or 1) is indicated in the legends of each graph. Log-rank *p*-values are provided in each panel. Figure S2. Kaplan–Meier drug survival curves stratified by MTX co-treatment status (0 = without MTX, 1 = with MTX) in switch patients for each bDMARD: (a) IFX, (b) ETN, (c) TCZ, (d) ADA, (e) ABT, (f) CZP, (g) GLM, and (h) SAR. MTX status (0 or 1) is indicated in the legends of each graph. Log-rank *p*-values are provided in each panel. Figure S3. Kaplan–Meier drug survival curves for each bDMARD in a sensitivity analysis wherein discontinuations due to hospital transfer were censored: (a) all 1182 patients, (b) 784 naïve patients, and (c) 398 switch patients. Log-rank *p*-values are provided in each panel. Table S1. Baseline characteristics of all patients (*n* = 1182). Table S2. Multivariable Cox proportional hazards model for treatment discontinuation in all patients, stratified by drug. Table S3. Multivariable Cox proportional hazards model for treatment discontinuation in switch patients, stratified by drug. Table S4. Detailed reasons for treatment discontinuation of each bDMARD in 1182 patients. Table S5. Multivariable Cox proportional hazards model for treatment discontinuation in all patients, stratified by drug, in a sensitivity analysis wherein hospital transfers were censored. Table S6. Multivariable Cox proportional hazards model for treatment discontinuation in naïve patients, stratified by drug, in a sensitivity analysis wherein hospital transfers were censored. Table S7. Multivariable Cox proportional hazards model for treatment discontinuation in switch patients, stratified by drug, in a sensitivity analysis wherein hospital transfers were censored.

## Abbreviations

The following abbreviations are used in this manuscript:

ABT	abatacept
ACR	American College of Rheumatology
ADA	adalimumab
bDMARDs	biological disease-modifying antirheumatic drugs
CZP	certolizumab pegol
ETN	etanercept
EULAR	EUropean League Against Rheumatism
GLM	golimumab
IFX	infliximab
MTX	methotrexate
NOSRAD	Niigata Orthopaedic Surgery Rheumatoid Arthritis Database
PSL	prednisolone
RA	rheumatoid arthritis
SAR	sarilumab
TCZ	tocilizumab
