# Supplementary material for: Five-Year Drug Survival and Discontinuation Reasons for Eight Biological Disease-Modifying Antirheumatic Drugs for Rheumatoid Arthritis: A Retrospective Analysis of 1182 Patients from the Niigata Orthopedic Surgery Rheumatoid Arthritis Database (NOSRAD)
Source: J Clin Med. 2026 Mar 9;15(5):2075. doi: 10.3390/jcm15052075 (PMC12985562; doi:10.3390/jcm15052075)
Supplement: Supplementary file 1 [file jcm-15-02075-s001.zip › jcm-4170668-supplementary.pdf]

Table S1. Baseline characteristics of all patients (n = 1,184)

	**IFX (*n* = 74)**	**ETN (*n* = 280)**	**TCZ (*n* = 390)**	**ADA (*n* = 86)**	**ABT (*n* = 145)**	**GLM (*n* = 95)**	**CZP (*n* = 53)**	**SAR (*n* = 59)**	**p-value**
Women, *n* (%)	57 (77.0)	236 (84.3)	305 (78.2)	60 (69.8)	115 (79.3)	77 (81.1)	44 (83.0)	43 (72.9)	0.106
Age (years), mean ± SD	55.6 ± 15.1	56.1 ± 15.4	57.6 ± 15.8	56.9 ± 13.7	70.3 ± 11.5	67.4 ± 13.5	56.5 ± 14.4	62.9 ± 14.3	<.0001
Disease duration (years), mean ± SD	9.5 ± 9.6	11.0 ± 9.6	9.1 ± 9.7	10.9 ± 9.8	13.3 ± 12.3	15.3 ± 11.1	8.9 ± 9.2	11.7 ± 8.5	<0.001
DAS28-ESR, mean ± SD	4.24 ± 1.3	3.83 ± 1.33	3.60 ± 1.39	3.30 ± 1.18	4.60 ± 1.36	2.96 ± 0.97	2.73 ± 1.14	3.58 ± 1.43	<0.001
MTX use, *n* (%)	69 (93.2)	177 (63.2)	181 (46.4)	67 (77.9)	54 (37.2)	69 (72.6)	30 (56.6)	25 (42.4)	<0.001
MTX dose (mg/week), mean ± SD	7.5 ± 1.9	7.3 ± 2.0	7.6 ± 2.3	8.0 ± 2.4	7.1 ± 2.9	7.5 ± 2.7	7.6 ± 2.2	6.9 ± 2.7	0.199
PSL use, *n* (%)	34 (46.0)	142 (50.7)	160 (41.0)	35 (40.7)	84 (57.9)	50 (52.6)	21 (39.6)	32 (54.2)	0.005
PSL dose (mg/day), mean ± SD	7.5 ± 8.8	6.6 ± 5.1	7.7 ± 7.1	5.1 ± 2.4	6.3 ± 5.1	4.5 ± 2.4	5.1 ± 2.4	5.4 ± 3.9	0.001
Naïve, *n* (%)	64 (86.5)	217 (77.5)	253 (64.9)	62 (72.1)	89 (61.4)	62 (65.3)	26 (49.1)	11 (18.6)	<0.001
Switch, *n* (%)	10 (13.5)	63 (22.5)	137 (35.1)	24 (27.9)	56 (38.6)	33 (34.7)	27 (50.9)	48 (81.4)	<0.001
2nd bio, *n* (%)	2 (2.7)	34 (12.1)	90 (23.1)	10 (11.6)	32 (22.1)	23 (24.2)	13 (24.5)	20 (33.9)	<0.001
3rd bio, *n* (%)	3 (4.1)	16 (5.7)	29 (7.4)	7 (8.1)	13 (9.0)	5 (5.3)	5 (9.4)	15 (25.4)	<0.001
≧4th bio, *n* (%)	5 (6.8)	13 (4.6)	18 (4.6)	7 (8.1)	11 (7.6)	5 (5.3)	9 (17.0)	13 (25.4)	<0.001

IFX, infliximab; ETN, etanercept; TCZ, tocilizumab; ADA, adalimumab; ABT, abatacept; GLM, golimumab; CZP, certolizumab pegol; SAR, sarilumab; DAS28-ESR, the 28-joint Disease Activity Score with erythrocyte sedimentation rate; MTX, methotrexate; PSL, prednisolone; bio, biologic agent; SD, standard deviation.

Table S2. Multivariable Cox proportional hazards model for treatment discontinuation in all patients, stratified by drug

Multivariable	HR	95% CI	p-value
Sex	1.25	0.97–1.61	0.084
Age	1.00	0.99–1.01	0.495
Disease duration	1.00	0.99–1.01	0.745
DAS28-ESR	0.91	0.84–0.98	0.015
MTX	0.86	0.69–1.10	0.202
PSL	1.22	0.99–1.49	0.059

p-values were derived from the multivariable Cox proportional hazards model, stratified by drug type. HR, hazard ratio; 95%CI, 95% confidence interval; DAS28-ESR, the 28-joint Disease Activity Score with erythrocyte sedimentation rate; MTX, methotrexate; PSL, prednisolone.

Table S3. Multivariable Cox proportional hazards model for treatment discontinuation in switch patients, stratified by drug.

Multivariable	HR	95% CI	p-value
Sex	0.89	0.56–1.42	0.634
Age	1.01	0.99–1.02	0.344
Disease duration	0.99	0.97–1.01	0.149
DAS28-ESR	0.99	0.86–1.14	0.920
MTX	1.06	0.68–1.68	0.789
PSL	1.15	0.79–1.67	0.478

p-values were derived from the multivariable Cox proportional hazards model, stratified by drug type. HR, hazard ratio; 95%CI, 95% confidence interval; DAS28-ESR, the 28-joint Disease Activity Score with erythrocyte sedimentation rate; MTX, methotrexate; PSL, prednisolone.

Table S4. Detailed reasons for treatment discontinuation of each bDMARDs in 1182 patients

	IFX (n = 74)	ETN (n = 280)	TCZ (n = 390)	ADA (n = 86)	ABT (n = 145)	GLM (n = 95)	CZP (n = 53)	SAR (n = 59)	p-value
Inadequate response	30 (40.5)	96 (34.3)	65 (16.7)	33 (38.4)	33 (22.8)	27 (28.4)	22 (41.5)	14 (23.7)	<0.001
Adverse events	15 (20.3)	56 (20.0)	65 (16.7)	15 (17.4)	19 (13.1)	16 (16.8)	13 (24.5)	5 (8.5)	0.256
Infections	3 (4.1)	27 (9.6)	29 (7.4)	3 (3.5)	8 (5.5)	4 (4.2)	5 (9.4)	0	0.082
Pulmonary disorders	0	5 (1.8)	3 (0.8)	1 (1.2)	3 (2.1)	0	0	0	0.495
Liver disorders	0	3 (1.1)	4 (1.0)	1 (1.2)	0	1 (1.1)	0	0	0.830
Skin disorders	2 (2.7)	1 (0.4)	9 (2.3)	7 (8.1)	1 (0.7)	5 (5.3)	1 (1.9)	2 (3.4)	0.002
Cardiovascular disease	0	1 (0.4)	4 (1.0)	0	2 (1.4)	0	0	0	0.633
Malignant tumor	3 (4.1)	2 (0.7)	3 (0.8)	1 (1.2)	1 (0.7)	3 (3.2)	2 (3.8)	0	0.084
Others	7 (9.5)	17 (6.1)	13 (3.3)	2 (2.3)	4 (2.8)	3 (3.2)	5 (9.4)	3 (5.1)	0.091
Remission/good response	2 (2.7)	15 (5.4)	14 (3.6)	6 (7.0)	1 (0.7)	2 (2.1)	2 (3.8)	1 (1.7)	0.180
Patient desire	0	10 (3.6)	13 (3.3)	0	6 (4.1)	2 (2.1)	1 (1.9)	1 (1.7)	0.409
Transfer to another hospital	15 (20.3)	64 (22.9)	47 (12.1)	5 (5.8)	13 (9.0)	16 (16.8)	2 (3.8)	5 (8.5)	<0.001
Other reasons	1 (1.4)	3 (1.1)	3 (0.8)	0	0	3 (3.2)	1 (1.9)	0	0.277
Unknown	4 (5.4)	4 (1.4)	22 (5.6)	5 (5.8)	7 (4.8)	1 (1.1)	1 (1.9)	1 (1.7)	0.072

Values are presented as *n* (percent).

bDMARDs, biological disease-modifying antirheumatic drugs; IFX, infliximab; ETN, etanercept; TCZ, tocilizumab; ADA, adalimumab; ABT, abatacept; GLM, golimumab; CZP, certolizumab pegol; SAR, sarilumab.

Table S5. Multivariable Cox proportional hazards model for treatment discontinuation in all patients, stratified by drug, in a sensitivity analysis wherein hospital transfers were censored

Multivariable	HR	95% CI	p-value
Sex	1.49	1.15–1.93	0.003
Age	1.00	0.99–1.01	0.507
Disease duration	1.00	0.99–1.01	0.823
DAS28-ESR	0.93	0.86–1.02	0.111
MTX	0.82	0.64–1.04	0.096
PSL	1.19	0.95–1.48	0.125

p-values were derived from the multivariable Cox proportional hazards model, stratified by drug type. HR, hazard ratio; 95%CI, 95% confidence interval; DAS28-ESR, the 28-joint Disease Activity Score with erythrocyte sedimentation rate; MTX, methotrexate; PSL, prednisolone.

Table S6. Multivariable Cox proportional hazards model for treatment discontinuation in naïve patients, stratified by drug, in a sensitivity analysis wherein hospital transfers were censored

Multivariable	HR	95% CI	p-value
Sex	1.54	1.09–2.17	0.013
Age	1.01	0.99–1.02	0.423
Disease duration	1.01	0.99–1.02	0.494
DAS28-ESR	0.94	0.85–1.05	0.261
MTX	0.69	0.50–0.94	0.019
PSL	1.12	0.84–1.49	0.427

p-values were derived from the multivariable Cox proportional hazards model, stratified by drug type. HR, hazard ratio; 95%CI, 95% confidence interval; DAS28-ESR, the 28-joint Disease Activity Score with erythrocyte sedimentation rate; MTX, methotrexate; PSL, prednisolone.

Table S7. Multivariable Cox proportional hazards model for treatment discontinuation in switch patients, stratified by drug, in a sensitivity analysis wherein hospital transfers were censored

Multivariable	HR	95% CI	p-value
Sex	0.85	0.54–1.33	0.465
Age	1.01	0.99–1.02	0.467
Disease duration	0.98	0.97–1.00	0.118
DAS28-ESR	0.96	0.84–1.10	0.561
MTX	1.13	0.74–1.73	0.561
PSL	1.17	0.81–1.69	0.393

p-values were derived from the multivariable Cox proportional hazards model, stratified by drug type. HR, hazard ratio; 95%CI, 95% confidence interval; DAS28-ESR, the 28-joint Disease Activity Score with erythrocyte sedimentation rate; MTX, methotrexate; PSL, prednisolone.

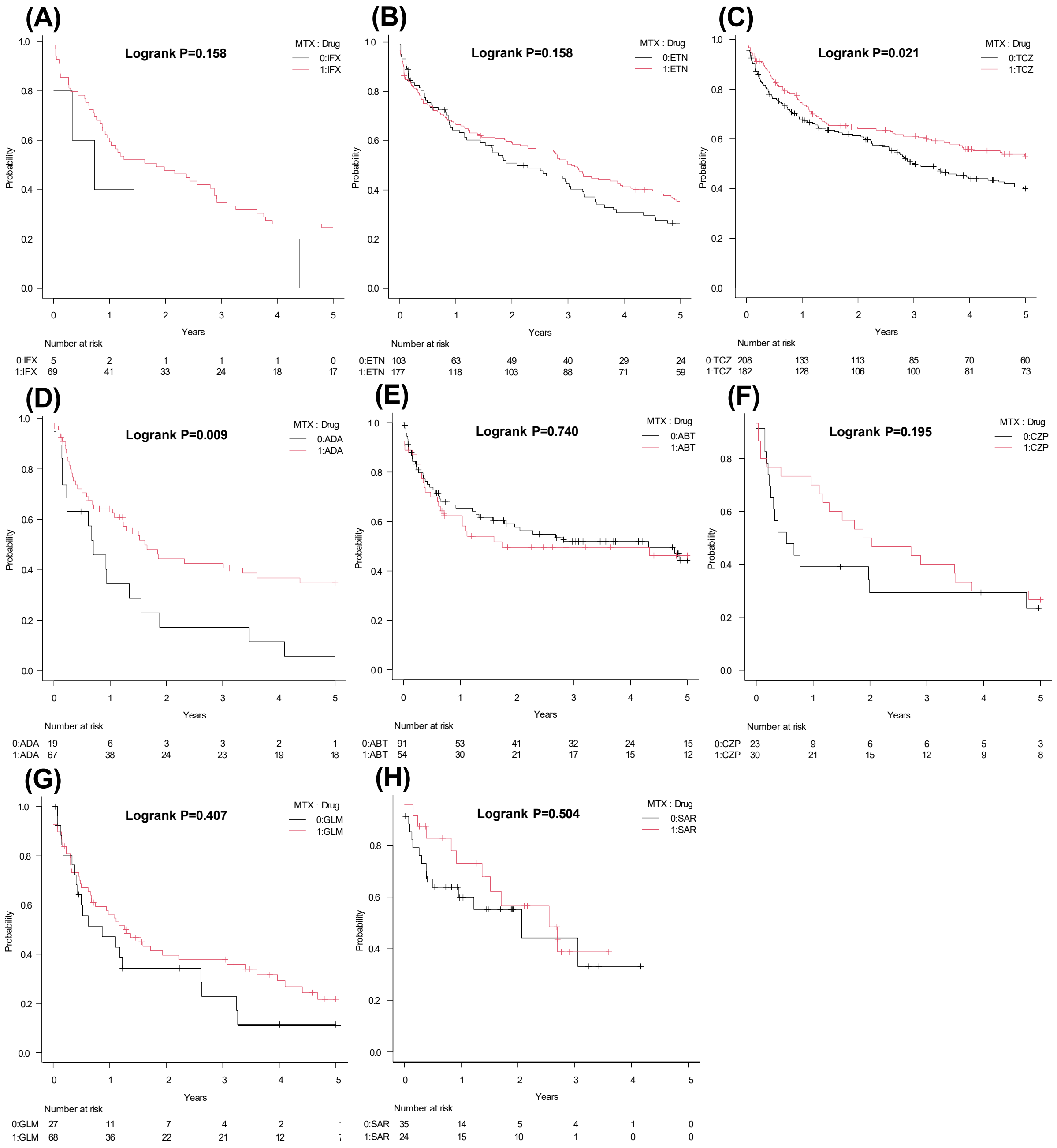

Figure S1. Kaplan–Meier drug survival curves stratified by MTX co-treatment status (0 = without MTX, 1 = with MTX) in all patients for each bDMARDs: (a) IFX, (b) ETN, (c) TCZ, (d) ADA, (e) ABT, (f) CZP, (g) GLM, and (h) SAR. MTX status (0 or 1) is indicated in the legends of each graph. Log-rank *p*-values are provided in each panel.

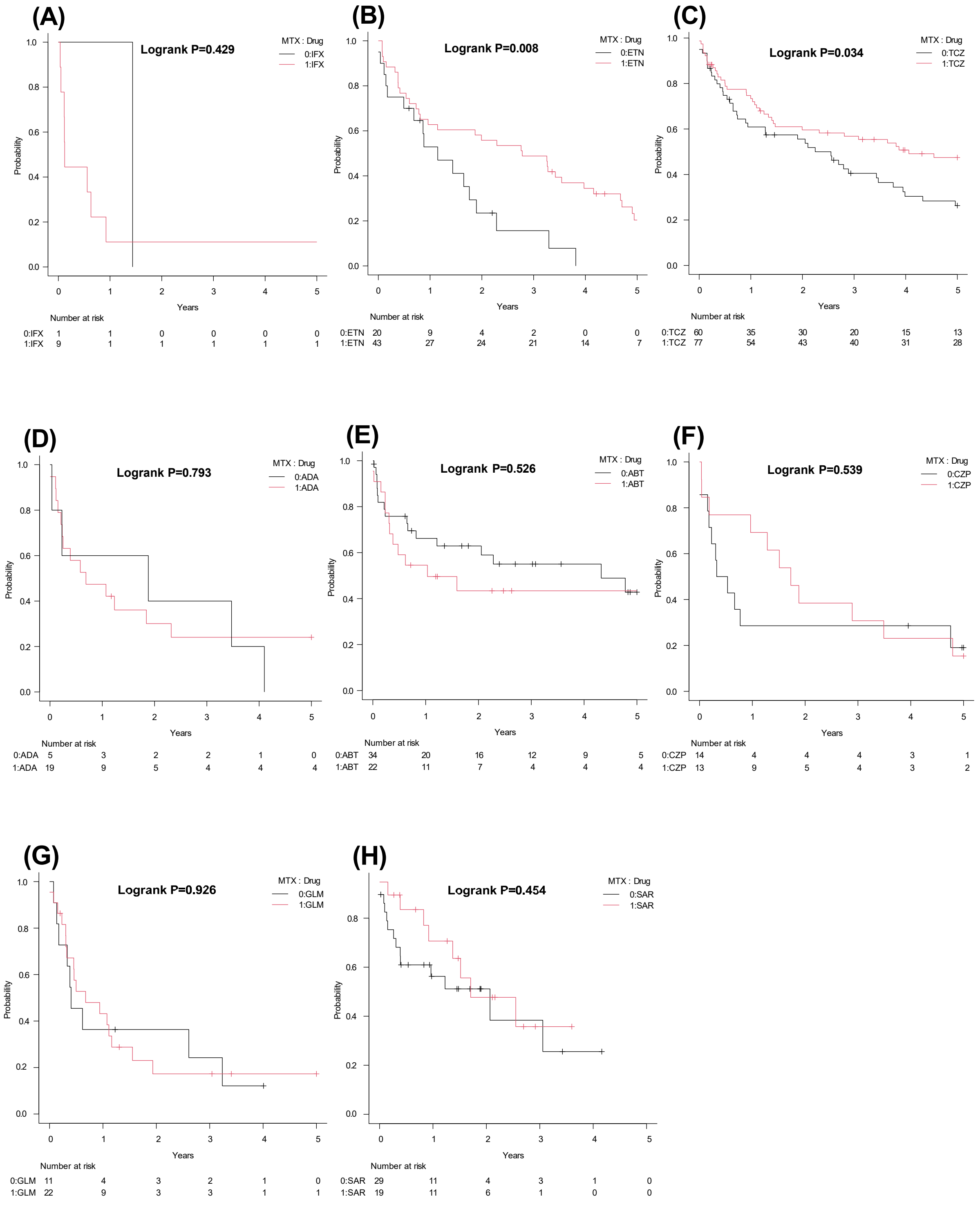

Figure S2. Kaplan–Meier drug survival curves stratified by MTX co-treatment status (0 = without MTX, 1 = with MTX) in switch patients for each bDMARDs: (a) IFX, (b) ETN, (c) TCZ, (d) ADA, (e) ABT, (f) CZP, (g) GLM, and (h) SAR. MTX status (0 or 1) is indicated in the legends of each graph. Log-rank *p*-values are provided in each panel.

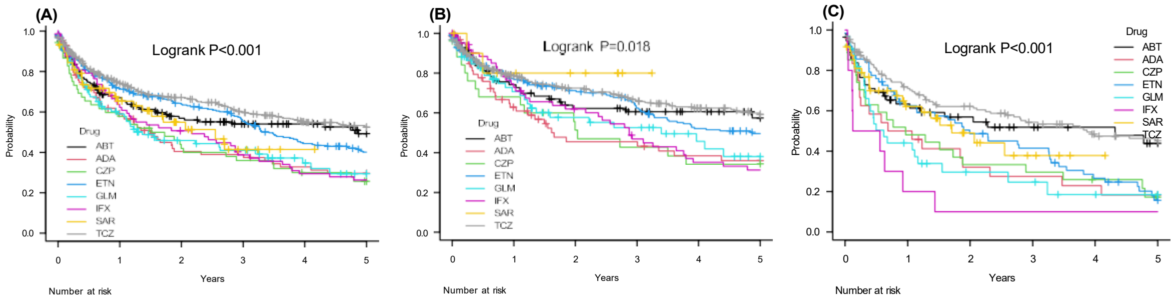

(A) Number at risk

	0	1	2	3	4	5
ABT	145	83	62	49	39	27
ADA	86	44	27	26	21	19
CZP	53	30	21	18	14	11
ETN	280	181	152	128	100	83
GLM	95	47	29	25	14	8
IFX	74	43	34	25	19	17
SAR	59	29	15	5	1	0
TCZ	390	261	219	185	151	133

(B) Number at risk

	0	1	2	3	4	5
ABT	89	52	39	33	26	18
ADA	62	32	20	20	16	15
CZP	26	17	12	10	8	8
ETN	217	145	124	105	86	76
GLM	62	34	23	20	12	7
IFX	64	41	33	24	18	16
SAR	11	7	5	1	0	0
TCZ	253	172	146	125	105	92

(C) Number at risk

	0	1	2	3	4	5
ABT	56	31	23	16	13	9
ADA	24	12	7	6	5	4
CZP	27	13	9	8	6	3
ETN	63	36	28	23	14	7
GLM	33	13	6	5	2	1
IFX	10	2	1	1	1	1
SAR	48	22	10	4	1	0
TCZ	137	89	73	60	46	41

Figure S3. Kaplan–Meier drug survival curves for each bDMARDs in a sensitivity analysis wherein discontinuations due to hospital transfer were censored: (a) all 1182 patients, (b) 784 naïve patients, and (c) 398 switch patients. Log-rank *p*-values are provided in each panel.
